# Advancing an Age-Friendly Ecosystem: Outside In and Inside Out

**DOI:** 10.1093/geroni/igaf122.1933

**Published:** 2025-12-31

**Authors:** Lisa Peters-Beumer

**Affiliations:** Concordia University Chicago, River Forest, Illinois, United States

## Abstract

Concordia University Chicago (CUC), an Age-Friendly University (AFU), participated in a 2020-2021 AFU study (Whitbourne et al., 2024) and subsequently was redesignated part of the AFU Global Network in 2023. Recommendations resulting from the study led the Center for Gerontology to spearhead the university’s AFU efforts and encompass a variety of initiatives in an effort to address ageism, increase awareness, and foster community engagement. Notably, the university held an Ageism Awareness Day (AAD) with a virtual event and campus-based booth, accompanied by a declaration from the CUC president. Additionally, CUC facilitated programs such as “Reframing Aging” with community partners, Careers in Aging Lunch and Learns, and Age Inclusive Higher Education in-person focus groups. Collaboration with leaders in this movement has enriched this work and driven internal age-inclusive prioritization discussions with campus constituents. These initiatives have garnered significant participation both in-person and virtually. Outcomes to date include increased awareness and growing engagement from faculty, staff, and students, with an increasing commitment to addressing ageism in academia. The focus of next steps includes enhancing the AFU ecosystem and its implications for future educational models that prioritize inclusivity and age diversity.

